# Improving Covariance Matrices Derived from Tiny Training Datasets for the Classification of Event-Related Potentials with Linear Discriminant Analysis

**DOI:** 10.1007/s12021-020-09501-8

**Published:** 2020-12-14

**Authors:** Jan Sosulski, Jan-Philipp Kemmer, Michael Tangermann

**Affiliations:** 1 Brain State Decoding Lab, Cluster of Excellence BrainLinks-BrainTools, Department of Computer Science, University of Freiburg, Freiburg, Germany; 2 University of Freiburg, Freiburg, Germany; 3 Autonomous Intelligent Systems Lab, Department of Computer Science, University of Freiburg, Freiburg, Germany; 4 Donders Institute for Brain, Cognition and Behaviour, Radboud University, Nijmegen, The Netherlands

**Keywords:** Event related potentials, Robust classification, Learning from small datasets, Noise transfer learning, Brain–computer interface, Covariance matrix enhancement

## Abstract

Electroencephalogram data used in the domain of brain–computer interfaces typically has subpar signal-to-noise ratio and data acquisition is expensive. An effective and commonly used classifier to discriminate event-related potentials is the linear discriminant analysis which, however, requires an estimate of the feature distribution. While this information is provided by the feature covariance matrix its large number of free parameters calls for regularization approaches like Ledoit–Wolf shrinkage. Assuming that the noise of event-related potential recordings is not time-locked, we propose to decouple the time component from the covariance matrix of event-related potential data in order to further improve the estimates of the covariance matrix for linear discriminant analysis. We compare three regularized variants thereof and a feature representation based on Riemannian geometry against our proposed novel linear discriminant analysis with time-decoupled covariance estimates. Extensive evaluations on 14 electroencephalogram datasets reveal, that the novel approach increases the classification performance by up to four percentage points for small training datasets, and gracefully converges to the performance of standard shrinkage-regularized LDA for large training datasets. Given these results, practitioners in this field should consider using our proposed time-decoupled covariance estimation when they apply linear discriminant analysis to classify event-related potentials, especially when few training data points are available.

## Introduction

A brain–computer interface (BCI) allows a subject to e.g. control a computer program using his or her brain signals, which are often recorded via the electroencephalogram (EEG), as it is non-invasive, requires relatively inexpensive equipment and could be used by a large part of the population (Wolpaw et al. 2002). Unfortunately, the signal-to-noise ratio of the signals recorded by the EEG electrodes on the scalp is bad, as many factors—e.g. volume conduction of the brain or the long distance between sensor and the brain tissue—impede the recording quality (Srinivasan 2012). To realize control via BCIs, machine learning techniques are key to decode the brain signals in real-time. In addition to the bad signal-to-noise ratio, the machine learning problem is aggravated by the oftentimes small amount of training data available in BCI experiments.

Existing approaches to deal with small datasets, such as transfer learning between subjects or sessions, have limited success if brain signals differ greatly between subjects and even between sessions of the same subject (Jayaram et al. 2016). Also, many BCI paradigms work optimally only, if their experimental parameters are tuned to each subject individually (Höhne and Tangermann 2012; Sugi et al. 2018; Allison and Pineda 2006). As many different experimental parameters need to be tested to find the optimal ones, the possibility to work with very small datasets would be a great benefit here.

The mentioned challenges explain the popularity of relatively simple classifiers in the BCI domain which can make efficient use of the training data (Blankertz et al. 2011). In contrast, in domains such as image recognition, the massive amounts of data available enable the employment of more sophisticated methods such as neural networks (Russakovsky et al. 2015).

**Fig. 1:**
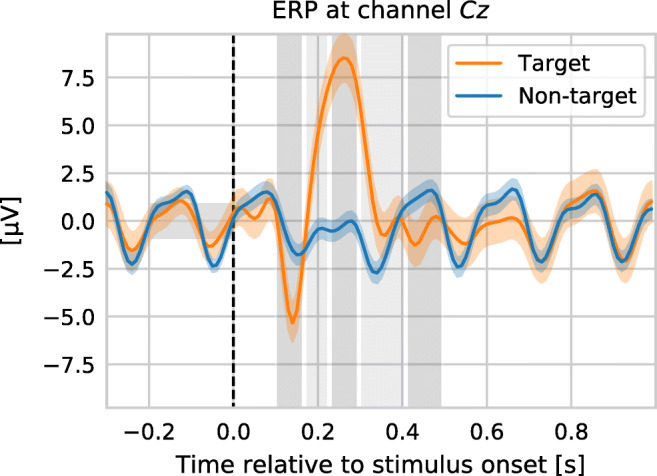
Example of the mean ERP responses obtained from a single subject during an auditory oddball paradigm with stimulus onset asynchrony (SOA) of 193 ms. For this plot, 300 target and 1500 non-target epochs were averaged. Prior to averaging, each epoch had been corrected for baseline shifts relative to the interval [-0.2, 0.0] seconds. Five gray shaded areas between 0.1 and 0.5 seconds post stimulus onset indicate which time intervals are typically used to derive features for classification

In this work, we are concerned with the classification of event-related potentials (ERPs) recorded using EEG data. These ERPs can be evoked by presenting visual, auditory or haptic stimuli to a subject (Sellers et al. 2012; Schreuder et al. 2010; Rutkowski and Mori 2015). Time-locked to the stimulus presentation, the ERP can be measured in the EEG signal. Due to the small ERP amplitude and the high amplitude of the EEG’s background activity, visualizations like in Fig. 1 require repeated stimulus presentations and averaging of the resulting ERP epochs. In this figure, two ERPs are shown, one for a specific stimulus the subject has attended (so-called target ERP), and another one for a single or even multiple other stimuli the subject has ignored (non-target ERP). The difference between target and non-target ERP voltages is the basis for classifying which stimulus a subject attends to in real-time. For a productive use of a BCI, however, it is infeasible to average such a large number of epochs before a classification output can be obtained. Therefore, machine learning is used to make classification possible on short recordings.

Many BCI systems make use of a linear discriminant analysis (LDA) (see e.g. Bishop 2006) to classify if a stimulus was attended or not. The LDA makes use of ERP voltage features and the corresponding covariance (Blankertz et al. 2011). In the domain of ERPs, the shrinkage-regularized LDA still belongs to the state-of-the-art methods (Lotte et al. 2007; Lotte et al. 2018). For ideal ERP data, the assumptions of LDA would even be fulfilled, making LDA the optimal classification approach. However, in practice there are non-stationarities, outliers and artifacts which violate the LDA’s assumptions. Recently, Riemannian methods found their way into BCI. For ERP classification they show promising performance gains for some datasets (Barachant et al. 2010; Barachant and Congedo 2014).

When using LDA to classify ERP signals, most formulations require an estimate of the covariance matrix. This matrix has 0.5 ⋅ (*D* + 1) ⋅ *D* free parameters with *D* being the dimensionality of the feature vector. Using an EEG cap with 31 channels and five time intervals only to derive voltage features (as indicated in Fig. 1), the feature dimensionality of *D* = 155 results in 12090 free parameters of the covariance matrix, which need to be estimated during the LDA training. Usually, the amount of data points (epochs) in BCI problems is rather small, leading to sub-optimal estimates of the covariance matrix. If the number of data points happens to be smaller than *D*, the covariance does not even have full rank and cannot be inverted.

In one of the example datasets in Blankertz et al. (2011) they found this to be especially true if “the number of training samples [is] low (750) compared to the dimensionality of the features (385)”. For comparison, in our benchmark the smallest training dataset has 72 training samples while the features have 310 dimensions. Farquhar and Hill (2013) show that classification performance increases with more training data, as they expected. However, they also note that “minimizing the number of training samples required to achieve acceptable performance is critically important to practical BCI performance”. Lotte et al. (2018) suggest that typical BCI systems could be trained with as few as 20-100 trials per class. In our benchmark, in the smallest dataset we use 12 target and 60 non-target training samples to train the classifiers. All authors recommend to shrink the covariance matrix used in LDA to the (scaled) unit sphere, especially when very few training data are available. The required amount of regularization can be determined analytically as proposed by Ledoit and Wolf (2004) or by using cross-validation.

There are alternative approaches for dealing with few training data. A first step is usually to perform strict data cleaning, such that the quality of the data is improved, for example using spatial filtering approaches (Winkler et al. 2014; Foodeh et al. 2016). Aside from regularization to deal with the dimensionality issue, another straightforward way is to reduce dimensionality altogether, e.g. by selecting channel subsets (see e.g. Lal et al.^2004^; Sannelli et al. 2010; Feess et al. 2013) or using preprocessing methods that find a lower-dimensional representation, e.g., xDAWN (Rivet et al. 2009) or kernel principal component analysis (kPCA) (Schölkopf et al. 1997). Other approaches employ transfer learning, which re-use data from previous sessions or even different subjects to improve the classification performance (Jayaram et al. 2016). However, in this work we focus on developing a method that can be applied without sophisticated preprocessing or using additional data. This both increases the employability of our method and facilitates the application to a large number of different ERP datasets.

In this work, we aim at improving the regularized covariance matrix even further by making use of the observation, that the *noise* in ERP data can be mostly attributed to task-unrelated background brain activity (Blankertz et al. 2011), which therefore is not time-locked to the stimulus.

To show the efficacy of our method named *time-decoupled covariance estimation*, we carefully evaluate its performance on datasets recorded by our lab as well as on public ERP datasets of which most are available in MOABB (Mother of All BCI Benchmarks) (Jayaram and Barachant 2018).

## Methods

We first describe our benchmarking approach. This includes the datasets we used, the general classification and validation procedure as well as the preprocessing of the EEG data. Afterwards we present the classification methods we use for comparisons. Finally, in “Time-decoupled Covariance Matrices” we detail our proposed new method with time-decoupled covariance estimation.

### Benchmark

To compare competing classification approaches, we evaluated their obtained performances on fourteen datasets, which have been derived from twelve ERP data sources (see Table 1) using MOABB (Mother of All BCI Benchmarks) (Jayaram and Barachant 2018). We used all (at the time of writing) ERP datasets available in MOABB and added complementing datasets from our lab, i.e. two additional visual speller datasets and ERP data from auditory paradigms with tone and word stimuli.

**Table 1:** Overview of the ERP datasets evaluated and their characteristics

Dataset	Subjects	Sessions	Channels	Paradigm	Access	Reference
EPFL_healthy*	4	4	32	visual, 6-choices	public	Hoffmann et al. (2008)
EPFL_patient*	4	4	32	visual, 6-choices	public	Hoffmann et al. (2008)
*BNCI_healthy_1*	10	1	16	visual, speller	public	Aricò et al. (2014)
*BNCI_healthy_2*	10	1	8	visual, speller	public	Guger et al. (2009)
*BNCI_patient*	8	1	8	visual, speller	public	Riccio et al. (2013)
*BI_a*^*+*^	7	8	16	visual, speller-like	public	Van Veen et al. (2019)
*BI_b*^*+*^	17	1	16	visual, speller-like	public	Van Veen et al. (2019)
*SPOT*	13	1	31	auditory, tones, oddball	public	Sosulski and Tangermann (2019)
*TONE_healthy*	20	1	63	auditory, tones, oddball	*closed*	Musso et al. (2016)
*WORD_healthy*	20	1	63	auditory, words, 6-choices	*closed*	Musso et al. (2016)
*TONE_patient*	14	11–25	31	auditory, tones, oddball	*closed*	*pending*
*WORD_patient*	10	11–25	31	auditory, words, 6-choice	*closed*	*pending*
*SPELLER_LLP*	12	1	31	visual speller	*closed*	Hübner et al. (2017)
*SPELLER_MIX*	12	1	31	visual speller	*closed*	Hübner et al. (2018)

Datasets listed as *closed* access have been recorded in our own lab but cannot be fully published as subjects’ consent had not been obtained for this purpose. Datasets marked with an asterisk and a plus sign each have been derived by splitting a larger data source (see main text)

For analysis purposes we have logically split two of the data sources into two datasets each: The original EPFL dataset (*EPFL*) was split into data obtained from healthy subjects and patients, while the brain invaders dataset (*BI*) was split into subjects with one session and subjects with eight sessions. Note that for the *TONE_patient* and the *WORD_patient* datasets, a publication is still pending, however the paradigms that were used are a tone oddball and a word oddball, similar to the paradigm described in Musso et al. (2016). Some datasets share subjects: the subjects who took part in the word paradigm in *WORD_healthy* also took part in the tone oddball paradigm in *TONE_healthy*. The same is true for *WORD_patient* and *TONE_patient*, except that in this case four additional subjects are contained in the latter dataset. For in-depth explanations of the datasets please refer to the corresponding references in Table 1. In total we evaluate on ERP data of 131 subjects (390 sessions in total).

We are primarily interested in the classification performance on very small datasets. However, some of the datasets available to us consist of many epochs per session of each subject. To investigate training concepts for small datasets, we have split most datasets into *virtual data subsets*. These splits were done at logical points in the paradigm. For example in the *SPOT* dataset, where a subject performed 60 to 80 auditory oddball runs with 90 stimuli each and an approximately eight second pause between runs, we could split this data up into virtual, non-overlapping subsets consisting of only 90 epochs, i.e. using each oddball run individually for a cross-validation loop. For this dataset, we therefore obtained between 60 and 80 virtual data subsets. For each novel subset, the classification performance was estimated in an individual cross-validation loop. In order to obtain a single classification performance value per session of a subject, we averaged the performances obtained from the virtual data subsets. Overall, the virtual data subsets have sizes between 90 and 4200 epochs.

### Evaluation Procedures

We used stratified 5-fold cross-validation within each virtual data subset in order to derive the classification performance expressed by the area under the receiver operating characteristic curve (AUC). Table 2 indicates for each dataset, how many epochs per session of each subject were available in total, and additionally, how many epochs were used within a virtual data subset.

**Table 2:** Average number of virtual data subsets (VDS) available for *a session of a subject* in each dataset

		# of Epochs
Dataset	# of VDS	per VDS
EPFL_healthy*	1	832
EPFL_patient*	1	833
*BNCI_healthy_1*	18	96
*BNCI_healthy_2*	1	2520
*BNCI_patient*	1	4200
*BI_a*^*+*^	1	480
*BI_b*^*+*^	1	480
*SPOT*	61	90
*TONE_healthy*	2	300
*WORD_healthy*	18	540
*TONE_patient*	2	300
*WORD_patient*	54	152
*SPELLER_LLP*	54	204
*SPELLER_MIX*	60	204

Also indicates the number of epochs within the virtual data subsets

Some datasets contained multiple sessions of each subject. We decided to condense them into a single AUC value per subject and classification approach. For this reason, AUC values obtained over multiple sessions of a subject were averaged before reporting.

Statistical significance (at *α* = 0.05) between the classification methods were determined using a paired Wilcoxon signed rank test (Wilcoxon et al. 1970) on the differences between the average performance of each dataset, i.e. 14 values. We compared our proposed method with its underlying base classification, and our proposed method with the best other classification method. Correction for multiple testing was done using the Holm–Bonferroni method (Holm 1979).

Using the code supplied in our repository,^1^ the benchmark results can be reproduced.

### Data Preprocessing

Before obtaining the actual features which can be used by the classifiers, all EEG datasets are preprocessed using a forward and a backward pass of a Butterworth bandpass filter in the range of 0.5 Hz to 16 Hz. Afterwards, the data was downsampled to 100 Hz and windowed to 0 s to 1 s relative to each stimulus onset to represent the corresponding data epoch.

**Fig. 2:**
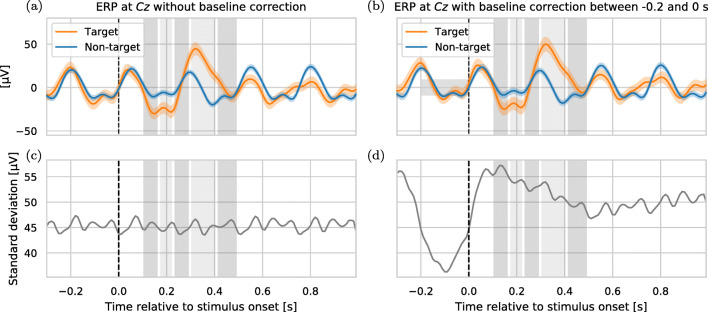
Top row: Averaged event-related potential of all epochs of subject 1 of the *BNCI_healthy_1* dataset in response to visual stimuli with two standard errors of the mean, **a** without and **b** with baseline correction as indicated by the horizontal gray box. Bottom row: Corresponding pooled standard deviation of the mean-free ERPs **c** without and **d** with baseline correction. As expected, the standard deviation is clearly reduced in the baseline interval. However, there is a distortion outside the baseline interval which leads to different standard deviations in the feature intervals (gray vertical boxes)

The common preprocessing step of baseline correction for ERP analysis causes the standard deviation in each channel to not be equal between time intervals (see Fig. 2), i.e. causes heteroscedasticity. As our proposed method assumes homoscedasticity, we both run the benchmark with and without baseline correction to determine the impact of this.

A common step to reduce the influence of artifacts is to exclude epochs that exceed a min-max criterion or reject channels that show abnormal variance. However, we kept all epochs and channels in all datasets, as picking the right criterion for each dataset can lead to subjective results. Using one common criterion for all datasets can also be detrimental, as the datasets recorded using visual paradigms tend to have large amplitudes compared to the auditory ones. Therefore, we consider the ability to cope with artifacts as another challenge for the evaluated classification methods.

A common preprocessing for LDA-based classifiers is to average the ERP responses in certain time intervals (cf. gray shaded areas in Fig. 1) to reduce the number of feature dimensions. When subject-specific maximized performance is desired, these time intervals could be determined automatically (see e.g. Bashashati et al. 2016). However, for comparability in our benchmark, we evaluated four sets of fixed time intervals (depending on the paradigm of the dataset, see Table 3) for all datasets and subjects for obtaining the features.

**Table 3:** Used time interval boundaries for the temporal sample-wise averaging for the different paradigms

# of Intervals	Interval boundaries [s]
Visual and tone paradigms	
2	{0.10, 0.18, 0.28}
5	{0.10, 0.17, 0.23, 0.30, 0.41, 0.50}
10	{0.10, 0.14, 0.17, 0.20, 0.23, 0.27,
	0.30, 0.35, 0.41, 0.45, 0.50}
40	$\{0.10, 0.11, \dots , 0.49, 0.50\}$
Word paradigms	
2	{0.40, 0.56},{0.65, 0.91}
5	{0.18, 0.26, 0.40, 0.56, 0.68, 0.91}
10	{0.18, 0.23, 0.29, 0.40, 0.48, 0.56,
	0.61, 0.68, 0.75, 0.82, 0.91}
73	$\{0.18, 0.19, \dots , 0.90, 0.91\}$

This averaging in time intervals is not necessary for the Riemannian method (cf. “Classification Methods”), as it uses xDAWN-preprocessing (Rivet et al. 2009), which extracts the ERP components in the whole epoch (0 s to 1 s) and inherently reduces dimensionality by using few obtained xDAWN components instead of the whole EEG channel set.

### Classification Methods

We employed three major types of classifiers. The first comprises three versions of the linear discriminant analysis, with each version using a different calculation method for the covariance matrix. All three versions directly use the EEG voltage features derived from sensor space. The number of voltage features per channel was treated as a hyperparameter. One version to calculate the covariance matrix for LDA is to estimate one matrix for each *class* and average these matrices into a common matrix. We call this LDA approach *LDA c-covs*. The implementation for this method was taken from the scikit-learn toolbox version v0.21.3 (Pedregosa et al. 2011). Alternatively, one can subtract the class-wise means from the data, and then *pool* the data of both classes and calculate one common covariance matrix from this pooled data (cf. (4) to (6) in “Feature Extraction and Covariance Calculation”). We refer to this approach as *LDA p-cov*. This was a custom implementation that can be found in our repository. The third version is the newly proposed LDA with a time-decoupled covariance estimation, named *LDA imp. p-cov*, which is detailed in “Time-decoupled Covariance Matrices”.

As we are interested in settings with tiny datasets, we often faced the situation that the feature dimensionality exceeds the number of training samples. Therefore, the second major classifier type uses a dimensionality reduction step, which was performed initially on the voltage features using a linear kernel PCA (Schölkopf et al. 1997). This results in a smaller number of component features, which were furthermore classified by a LDA c-covs approach. The number of components to use was treated as a hyperparameter. In each cross-validation split, the kPCA components were calculated on the training fold and applied to both training and test folds. For brevity, we refer to this classifier type as *kPCA*.

The third classifier type makes use of a specific space to represent each epoch as a covariance matrix and perform operations in this space of covariance matrices using Riemannian geometry. First proposed for BCI by Barachant and colleagues for motor imagery data (Barachant et al. 2010), extensions for ERP processing have been proposed. We followed the ERP analysis pipeline of Kolkhorst et al. (2018), making use of xDAWN as a spatial filter preprocessing step (Rivet et al. 2009), extending the feature representation by target (and non-target) templates prior to calculating the covariance matrix per epoch, and a classification thereof in a tangent space representation using logistic regression. Note that in each cross-validation split, the xDAWN components were determined on the training fold and applied to both training and test folds. For the Riemannian method, we varied the number of xDAWN components between one and six and treated this choice as a hyperparameter. Additionally we varied whether the target class only, or both target and non-target class templates were used in the covariance representation. This pipeline will be referred to as *Riemann*.

For all classifier types, hyperparameters were evaluated using values from a predetermined grid. In the case of the LDA types and kPCA, the boundaries for the time interval features considered are given by Table 3. For instance, the boundaries {0.10,0.18,0.28} describe two time intervals, with the first being [0.10,0.18) and the second one [0.18,0.28).

All evaluated hyperparameters can be found in Table 4. For the kPCA and the Riemann methods, all possible hyperparameter combinations are evaluated. To avoid overfitting, we report the single parameter set which obtained the best average performance across all datasets and subjects.

**Table 4:** All evaluated hyperparameters for each type of classifier

Type	Hyperparameter	Values
LDA	Time intervals	{2, 5, 10,all}
kPCA	Time intervals	{2, 5, 10,all}
	kPCA comps.	$\{10,20,\dots ,90,\text {all}\}$
Riemann	xDAWN comps.	{1, 2, 3, 4, 5, 6}
	Template class	{both,target}

A value of ‘all’ time intervals means that every EEG sample in the ERP interval is taken individually, the exact time points differ between the paradigms (cf. Table 3)

### Feature Extraction and Covariance Calculation

This section details the typical process of obtaining amplitude-based features and the LDA weights as detailed by Blankertz et al. (2011) for ERP-based BCIs. We describe this procedure very detailed, as we build upon parts of it in the next section for our proposed method.

The number of epochs per training dataset, the number of available EEG channels and the number of time intervals (or, when using kPCA preprocessing, the number of kPCA components) varied between datasets. However, for readability we will simplify the notation in this section and the next, by providing the formulae for an example dataset with 31 channels, 5 time intervals per channel and 90 training epochs. Note however, that the method can be applied to any number of channels, epochs or time intervals larger than one.

We use the notation $x_{c_{i}}^{T_{j}}$ for the scalar value representing the voltage in the *i*-th channel *c* during the *j*-th time interval *T* of one epoch. This yields the stacked feature vector

1 $$  \boldsymbol{x} := ({x}^{c_{1}}_{T_{1}}, {x}^{c_{2}}_{T_{1}}, \dotsc, {x}^{c_{31}}_{T_{1}}, {x}^{c_{1}}_{T_{2}}, \dotsc, {x}^{c_{31}}_{T_{5}} )^{\mathsf{T}}, $$

which contains the relevant voltage features of a single epoch, with $\boldsymbol {x} \in \mathbb {R}^{155}$. Stacking the feature vectors ***x*** of all 90 available epochs of a single trial, we obtain the data matrix

2 $$  X := [\boldsymbol{x}_{1}, \boldsymbol{x}_{2}, \dotsc, \boldsymbol{x}_{90}], $$

with $X \in \mathbb {R}^{155 \times 90}$ and ***x***_*i*_ belonging to the *i*-th epoch.

Similarly, the class labels of all 90 epochs are contained in the vector

3 $$  \boldsymbol{y} := (y_{1}, y_{2}, \dotsc, y_{90})^{\mathsf{T}} \quad y_{i} \in \{0, 1\}, $$

with an entry of 1 indicating a target stimulus and 0 a non-target of the *i*-th epoch.

Before calculating the covariance matrix *Σ*, we must make *X* mean-free. As we have two different classes in our data, target and non-target, we need the class-wise means

4 $$  {M}_{i} := \begin{cases} \boldsymbol{\mu}_{1} & \quad \text{if } y_{i} = 1 \\ \boldsymbol{\mu}_{0} & \quad \text{if } y_{i} = 0 \\ \end{cases}, $$

where *M*_*i*_ describes the *i*-th column of the matrix *M*, with ***μ***_1_ and ***μ***_0_ containing the average target / non-target ERP voltages (in these 90 epochs), respectively. Now we can calculate the class-wise mean-free feature matrix

5 $$  \widetilde{X} := X-M, $$

and finally obtain the sample covariance matrix

6 $$  \hat{{\varSigma}} := \frac{1}{N-1} \widetilde{X}\widetilde{X}^{\mathsf{T}}, $$

with $\hat {{\varSigma }} \in \Bbb R^{155 \times 155}$. Given that in this example we consider using only 90 epochs, $\hat {{\varSigma }}$ is linearly dependent and therefore not invertible. In addition, $\hat {{\varSigma }}$ badly approximates the true underlying covariance matrix *Σ* due to a systematic bias of overestimating large and underestimating small eigenvalues when too few datapoints are available (Blankertz et al. 2011). A practical method to obtain an invertible covariance matrix and counter the abovementioned bias is to regularize the covariance matrix toward the main diagonal:

7 $$ \begin{array}{@{}rcl@{}} \nu &:=& \text{diag}(\hat{{\varSigma}}) \end{array} $$

8 $$ \begin{array}{@{}rcl@{}} \widetilde{{\varSigma}} &:=& (1-\gamma)\hat{{\varSigma}} + \gamma \bar{\nu} I \end{array} $$

The sample covariance matrix $\hat {{\varSigma }}$ is regularized towards a diagonal matrix where diagonal entries correspond to the average $\bar {\nu }$ of the diagonal values of $\hat {{\varSigma }}$. The regularization strength *γ* was obtained using the Ledoit–Wolf lemma (Ledoit and Wolf 2004).

### Time-decoupled Covariance Matrices

Our proposed method builds on the general LDA p-cov pipeline from the previous section, but improves the covariance matrix by a better estimation of the spatial noise structure. This is made possible by time-decoupling of the noise estimation.

For the purpose of classification using LDA, two common domain-specific assumptions about the noise in ERP data work well in practice (Blankertz et al. 2011): The first (*A1*) states that the noise on the ERP features is normally distributed and has zero-mean, which is reasonable to assume when using a high-pass filter on the measured signal and acknowledging that the EEG background noise is the result of many spatio-temporally overlapping brain sources. The second assumption (*A2*) is that the noise is unrelated to the current user task (i.e. either attend or ignore a stimulus) or—going one step further—if a stimulus has been played recently or not. On the level of a single epoch, this means that within a single EEG channel the noise should be homoscedastic, i.e. the same for the five extracted voltage features per channel. We saw before, that this is approximately fulfilled when no baseline correction is performed on the epochs (cf. Fig. 2). For the most common noise sources, such as technical noise and background EEG activity this assumption seems reasonable.

In the conventional estimation of the covariance matrix, the channel-wise noise within a time interval is estimated for each time interval individually. However, when there is no difference in the channel-wise noise between the time intervals (*A2*), it seems reasonable to estimate one common channel-wise covariance matrix that is decoupled from the different time intervals. We propose the idea, to obtain a robust estimation of the between-channel covariance matrix in order to enhance the covariance matrix needed for the calculation of the LDA weight vectors and bias. We thereby build on the process described in “Feature Extraction and Covariance Calculation”.

The dimensionality of the feature vector ***x*** results in a covariance matrix $\widetilde {{\varSigma }}$ with a particular structure. In order to describe submatrices of $\widetilde {{\varSigma }}$ we use the notation $\widetilde {{\varSigma }}_{i:j, n:m}$ which indicates the submatrix obtained by using the *i*-th up to the *j*-th row and the *n*-th up to the *m*-th column from $\widetilde {{\varSigma }}$. For example, using the feature vector definition as described in Eq. 1, the matrix $\widetilde {{\varSigma }}_{1:31,1:31}$ would describe the covariance between all 31 channels within the first time interval *T*_1_. The covariance between all channels and between time intervals *T*_1_ and *T*_2_ is contained in $\widetilde {{\varSigma }}_{1:31, 32:62}$ (and $\widetilde {{\varSigma }}_{32:62, 1:31}$ the other way around).

If *A2* is true, the covariance between channels (given time intervals of the same size) should look similar within each time interval, i.e.,

9 $$ \widetilde{{\varSigma}}_{1:31, 1:31} \simeq \widetilde{{\varSigma}}_{32:62, 32:62} \simeq \dotsc \simeq \widetilde{{\varSigma}}_{125:155, 125:155} $$

These five different blocks, which describe the covariance between channels separately for the five time intervals will be called $B_{1}, B_{2}, \dotsc , B_{5}$ in the following.

**Fig. 3:**
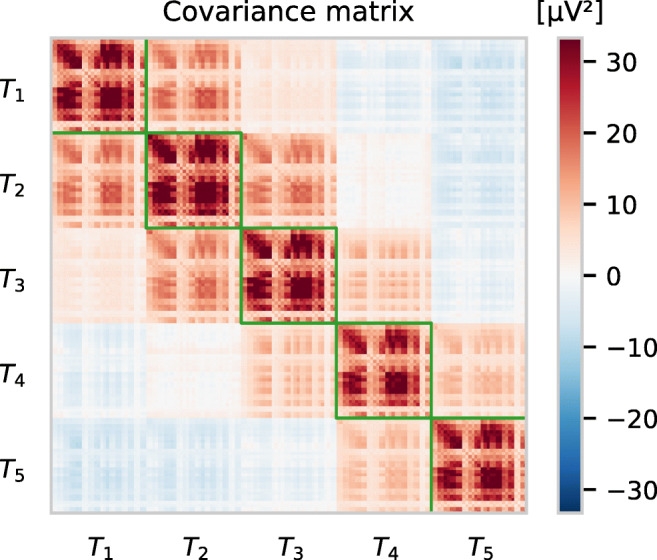
Covariance matrix of the ERP features depicted in the ERP plot in Fig. 1, except that no baseline correction was applied (see Fig. 2). There are five distinct blocks (indicated by green borders), each containing the covariance between EEG channels within one time interval

An example of a covariance matrix obtained from ERP data is given by Fig. 3 as a heat map. The within-time interval blocks on the main diagonal (depicted with a green border) show a similar structure, but slightly vary in the average intensities. The latter is caused by a different number of temporal samples averaged in each time interval. However, if both *A1* and *A2* are true, the within-time interval channel covariance matrices *B*_*m*_ should (aside from noise) be equal, if the number of samples that are averaged in each of the time intervals *T*_*m*_ are identical. In this case, we can calculate the within channel covariance regardless of a specific time interval in the epoch. Let

10 $$  \boldsymbol{x}^{C} := ({x}^{c_{1}}, {x}^{c_{2}}, \dotsc, {x}^{c_{31}})^{\mathsf{T}}, $$

with $\boldsymbol {x}^{C} \in \mathbb {R}^{31}$ represent the features of a single time interval only. For our example, we obtain five of these vectors ***x***^*C*^ per epoch, one for each of the five different time intervals. Stacking these vectors, the feature matrix can be re-arranged to

11 $$ X^{C} := [\boldsymbol{x}^{C}_{1}, \boldsymbol{x}^{C}_{2}, \dotsc, \boldsymbol{x}^{C}_{450}], $$

with $X_{C} \in \mathbb {R}^{31\times (90\cdot 5)}$. Compared to Eq. 2, we now have a much larger number of samples to estimate this smaller between-channel covariance matrix $\hat {{\varSigma }}^{C}$. The calculation of $\hat {{\varSigma }}^{C}$ can be performed as described in Eqs. 5 and 6. Empirically we found, however, that shrinkage regularization should be avoided (except when *D* > *N*) as it negatively affects classification performance. Note, that if the width of the feature time intervals ${T_{1}, T_{2}, \dots , T_{5}}$ differs, the data should be scaled to a common variance prior to creating *X*^*C*^. This can be accomplished by considering the number of samples in a time interval *T*_*m*_ averaged per time interval, leading to a scaling factor of $\sqrt {|T_{m}|}$ for the *m*-th time interval.

After obtaining an estimate for the between-channel covariance matrix $\hat {{\varSigma }}^{C}$, we use it to replace the blocks *B*_*m*_ of the whole covariance matrix $\hat {{\varSigma }}$, however, only after having rescaled $\hat {{\varSigma }}^{C}$ to match the determinant of *B*_*m*_, i.e.

12 $$  \hat{{\varSigma}}^{C}_{m} := \left( \frac{\det B_{m}}{\det \hat{{\varSigma}}^{C}}\right)^{\frac{1}{31}} \hat{{\varSigma}}^{C}. $$

This rescaling ensures $\det B_{m} = \det \hat {{\varSigma }}^{C}_{m}$. Intuitively, the rescaling has the effect, that the overall spread of the data distribution described by the covariance matrix $\hat {{\varSigma }}^{C}_{m}$ remains equal to the overall spread of the data distribution described by *B*_*m*_.

After the rescaling, we can substitute *B*_*m*_ with $\hat {{\varSigma }}^{C}_{m}$ and obtain a new covariance matrix $\dot {{\varSigma }}$ which will be used for the calculation of the classifier weights in linear discriminant analysis:

13 $$ \begin{array}{@{}rcl@{}} \boldsymbol{w} &:= \dot{{\varSigma}}^{-1}(\boldsymbol{\mu}_{\boldsymbol{0}} - \boldsymbol{\mu}_{\boldsymbol{1}}) \end{array} $$

14 $$ \begin{array}{@{}rcl@{}} b &:= \frac{1}{2}\boldsymbol{w}(\boldsymbol{\mu}_{\boldsymbol{0}} + \boldsymbol{\mu}_{\boldsymbol{1}}). \end{array} $$

This new covariance matrix $\dot {{\varSigma }}$ can be understood as a covariance matrix in which the blocks on the main diagonal, i.e. the between-channel covariance, has been time-decoupled. Hereinafter we refer to the LDA that uses this way of time-decoupling to improve the pooled data covariance matrix as *LDA imp. p-cov*.

## Results

We first report the average performance of the tested classification methods on all datasets and then the influence of training dataset size on performance differences between the methods. Finally, subject-wise results are shown for some datasets.

### Optimal Hyperparameters and Grand Average Performance

Searching through the hyperparameter space, we found that on average ten time intervals were optimal for all LDA-based approaches. For kPCA preprocessing, 70 components performed best across the datasets. The Riemannian-based classifier obtained the best performance when using only the target class as a template and using five xDAWN components (see Table 5).

**Table 5:** Optimal hyperparameters that produced the best performance on average across all datasets for each classification method

Method	Hyperparameter	Value
LDA imp. p-cov	Time intervals	10
LDA p-cov	Time intervals	10
LDA c-covs	Time intervals	10
kPCA	Time intervals	10
	kPCA components	70
Riemann	Template class	Target
	xDAWN components	5

**Fig. 4:**
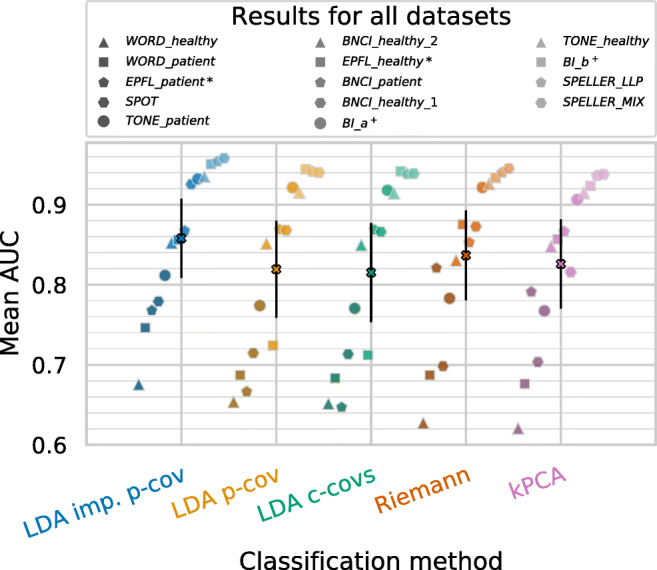
Performances for all datasets individually (polygon markers) and averaged (‘X’ markers), both after averaging across subjects. Black error bars indicate two standard errors of the mean

The grand average results using these hyperparameters are shown as black markers in Fig. 4. Colored markers indicate the average AUC values across subjects separately for each dataset. The proposed new LDA method (LDA imp. p-cov) using the time-decoupled pooled covariance matrix outperformed the corresponding LDA method (LDA p-cov) with a standard shrinkage-regularized pooled covariance matrix by about 4 % points AUC (*p* = 0.003). The kPCA is supposed to handle large feature dimensionalities rather well. As its performances, however, are very close to those of the LDA p-cov, we assume that the improvement of our proposed novel method is not merely caused by a better handling of high-dimensional data.

The AUC improvement of our novel approach is still around 2 % AUC points on average (*p* = 0.036) when comparing the proposed novel approach with the runner up, the Riemannian method.

We observed strong discontinuities in the raw data of the *EPFL* datasets, expressed by sudden step-wise voltage offsets in the data. This seems to cause serious problems for the LDA-based methods, whereas the Riemannian method copes better with these discontinuities. Applied to the non-*EPFL* datasets, the Riemann method does not show a clear advantage over the LDA methods (see Fig. 4). The reported advantage of Riemannian methods on ERP data (cf. Kolkhorst et al. 2018; Barachant and Congedo 2014) may be more pronounced on larger training datasets. These, however, were rare in our benchmark (median: 300 epochs, inter-quartile range: 204 to 540 epochs).

Interestingly, kPCA increases the average performance of the LDA with class-wise covariance matrices. However, the effect is not very large. Figure 4 reveals that kPCA improves performance greatly for the *EPFL* datasets compared to LDA c-covs, but for most other datasets its performance decreases slightly. This indicates kPCA’s ability to deal well with the discontinuities in the *EPFL* datasets. Lower performances on the remaining datasets indicate that kPCA’s hyperparameters do not generalize well over all datasets.

The effect of baseline correction on the classification performance of the different classifiers is provided by Table 6. We found that the performance of LDA classifiers tends to decrease when using a baseline interval of -0.2 s to 0 s compared to using no baseline correction at all. A possible explanation for this is the effect baseline correction has on the feature’s standard deviations as shown in Fig. 2. As applying baseline correction violates assumption *A2* (cf. “Time-decoupled Covariance Matrices”) the performance decay of LDA imp. p-cov is largest among all methods. The Riemannian classification method is the only one that benefits marginally (0.003 AUC points) from baseline corrections. Note that we used a high-pass with a threshold of 0.5 Hz. In the case of even lower thresholds, the influence of baseline correction may have to be re-evaluated.

**Table 6:** Comparison of the grand average AUC performances across all datasets and subjects of the evaluated classification methods

	Baseline correction:	
Method	No	Yes
LDA imp. p-cov	**0.858**	0.818
LDA p-cov	**0.819**	0.811
LDA c-covs	**0.815**	0.808
Riemann	0.836	**0.839**
kPCA	**0.826**	0.815

This table shows the detrimental effects of baseline corrections on LDA classification performance

### Influence of Training Dataset Size

**Fig. 5:**
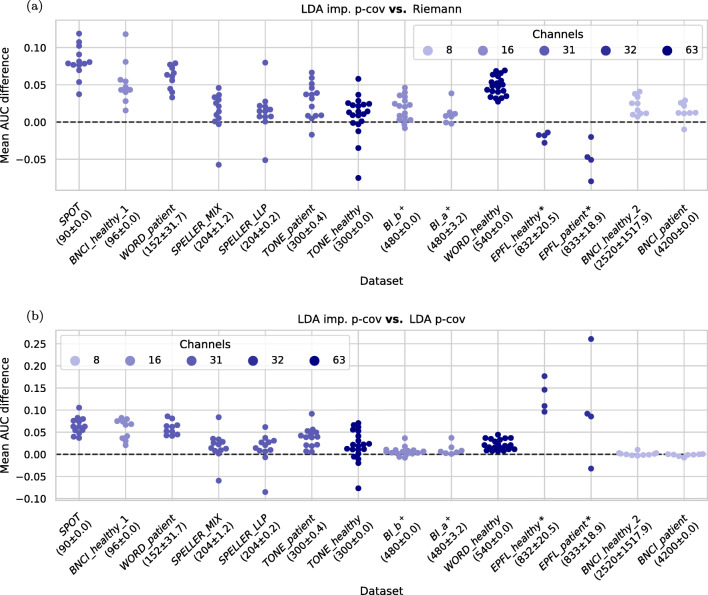
**a** Separately for each subject and dataset, the mean AUC difference between the LDA using the time-decoupled pooled covariance and the Riemannian method is provided. **b** Corresponding AUC differences of the new method in comparison with an LDA using pooled covariance matrices (bottom). In this overview, the Riemannian methods use the overall optimal hyperparameters (five xDawn components, only target class templates) for each dataset and the LDAs use the overall optimal number of ten time intervals for each dataset. From the left to right the datasets are ordered by the average number of epochs in a virtual data subset (mean and standard deviation are provided in brackets). Colors encode the number of EEG channels in a dataset

In the top plot in Fig. 5a the performance difference between the proposed LDA imp. p-cov and the runner-up Riemann is shown for each subject and dataset. The proposed method outperforms the Riemann method especially when the amount of training data is small but it stays marginally superior also for most larger datasets. The *EPFL* datasets deviate from this observation, which could be attributed to the Riemann method’s ability to cope well with artifacts, as these datasets contain strong discontinuities in the epoched signals.

We observe, that the Riemann method performs particularly bad on the relatively large *WORD_healthy* dataset. In this dataset, the informative ERP features tend to have larger latencies than in datasets using less complex stimuli.

For three subjects the performance is more than 5 % points AUC worse when using the LDA imp. p-cov method. They belong to the datasets *SPELLER_MIX*, *SPELLER_LLP* and *TONE_healthy*. Closer investigation revealed that for some virtual data subsets in these subjects the eigenvalues of the covariance matrix were no longer all positive after replacing the diagonal blocks, causing the poor average performance when employed in the LDA.

Figure 5b shows how the LDA imp. p-cov method compares to the regular LDA p-cov method. Here, the same trend with respect to virtual data subset sizes can be observed. Interestingly, our proposed method seems to handle the discontinuities present in the *EPFL* datasets much better, leading to large performance differences between these two methods. Compared to the Riemann method, we can now see that the performances are nearly equal for the two largest datasets positioned on the right end of the horizontal axis.

**Fig. 6:**
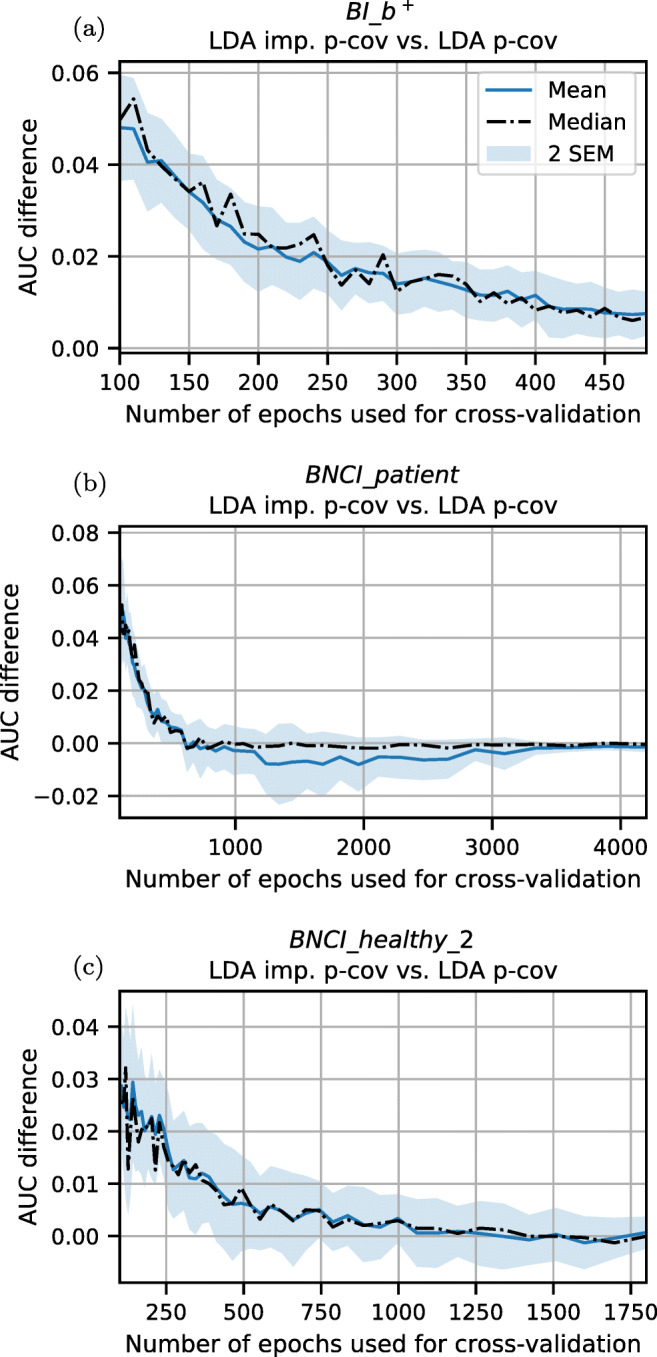
Impact of increasing training samples on the performance difference between LDA imp. p-cov and LDA p-cov for the datasets **a** *BI_b*^*+*^, **b** *BNCI_patient* and **c** *BNCI_healthy_2*. Values above 0 indicate a better performance of our proposed LDA imp. p-cov method. With growing dataset sizes, both methods converge to the same performance

The impact of having few training data depends on multiple factors, such as paradigm, signal-to-noise ratio and dimensionality. To better quantify this impact, we additionally evaluated how the performance difference between our proposed method and the baseline LDA p-cov method develops depending on the amount of training data. For three datasets, we trained both classifiers using an increasing number of training samples per VDS from 100 up to the largest amount that was available for all subjects in a dataset. In order to obtain standard error estimates, we calculated the cross-validated AUC for each VDS size 20 times on different within-class permutations. As shown in Fig. 6, for few training samples LDA imp. p-cov provides a better average performance for each dataset. As expected, this improvement is reduced when more and more training data is used. Note that for the *BNCI_patient* dataset (Fig. 6b), the mean drops below 0, while the median stays close to 0. This difference is caused by only one subject who has bad performance using the LDA imp. p-cov with 1000 to 3000 epochs for the cross-validation.

**Fig. 7:**
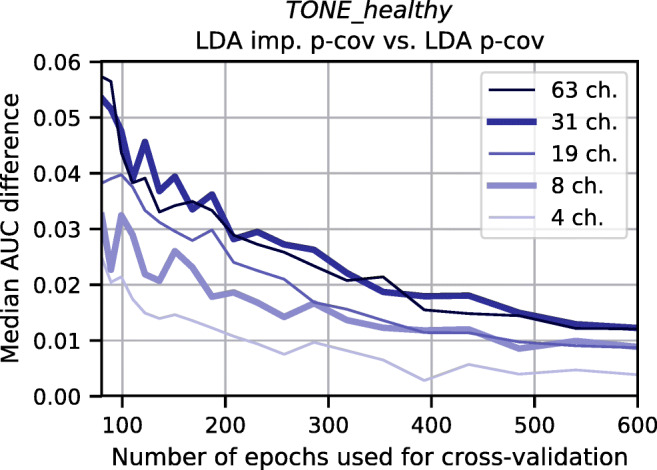
Interaction between amount of training data and number of channels. Each curve represents a different number of channels and depicts the performance difference between LDA imp. p-cov and LDA p-cov for the *TONE_healthy* dataset for varying number of training samples per VDS. For this purpose, the full channel set of 63 channels was reduced to smaller, approximately equidistant sets. Each curve provides the median of 20 permutations. Values above 0 indicate a better performance of our proposed LDA imp. p-cov method

Our proposed method estimates a more reliable version of the between-channel covariance matrix. To determine how the number of channels impacts our proposed method, we evaluated on the *TONE_healthy* dataset (as it offered the largest number of channels) with both increasing number of data for the VDS as well as with artificially reduced channel subsets. The results in Fig. 7 show that the performance improvement remains relatively stable when using 63 or 31 channels. However, especially when using very small channel sets of only four channels, the performance improvement obtained by our new approach is lower across all training data set sizes, when compared to using the full set of channels.

### Subject-wise Results for Selected Datasets

**Fig. 8:**
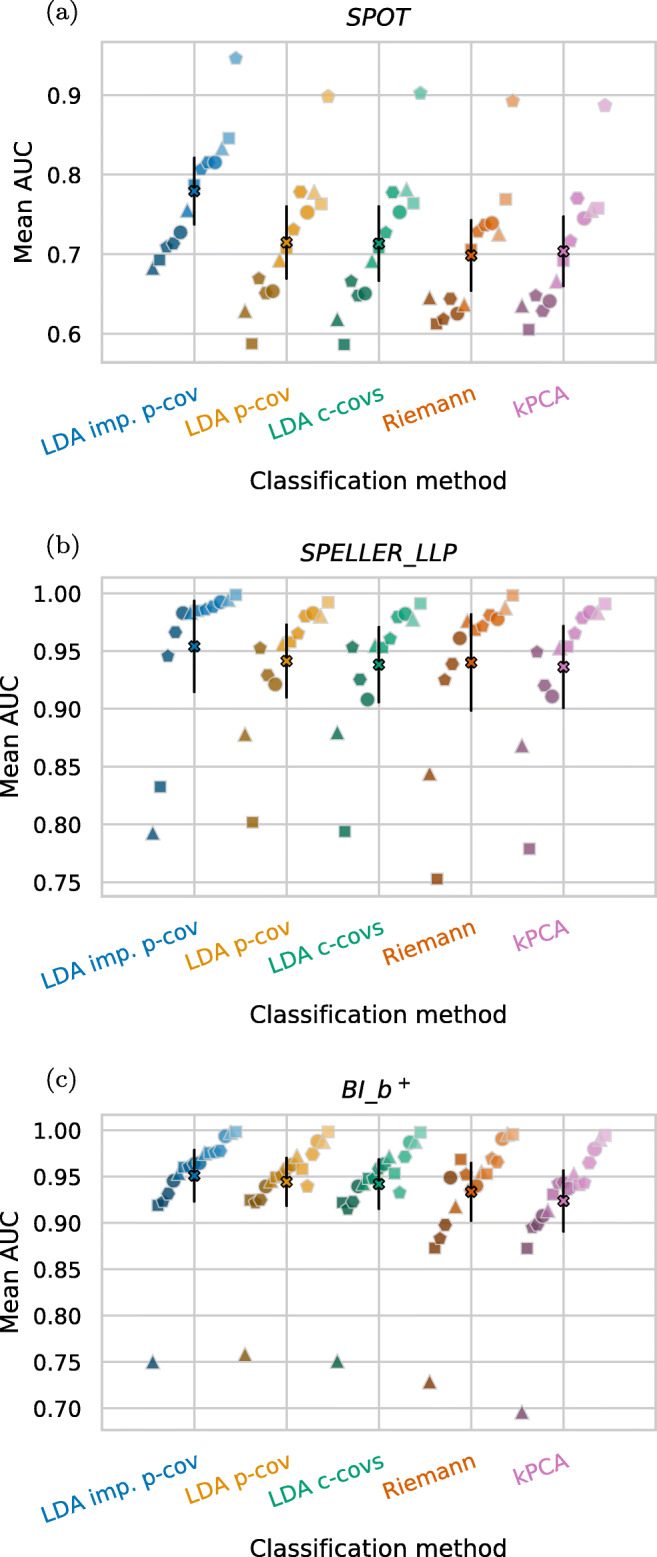
Results for the **a** *SPOT*, the **b** *SPELLER_LLP* and the **c** *BI_b*^*+*^ dataset. Subjects can be matched by marker type and color brightness. Within one classification method strip, subjects are ordered from left to right with respect to their individual scores obtained by the LDA imp. p-cov method. The cross marker indicates the mean AUC, with black bars marking two standard errors of the mean

In Fig. 8, absolute AUC performances of each subject are provided for three selected datasets and separately for the five classification approaches.

The *SPOT* dataset on top (a) has the smallest virtual data subsets of 90 epochs each. We can see that our proposed method outperforms all other methods for every individual subject. Additionally, the ranking between subjects is very stable between the five approaches and specifically between the LDA-based methods.

In Fig. 8b, the *SPELLER_LLP* dataset with 204 epochs per virtual data subset is shown. A few outlier subjects are observed with markedly decreased performances. The left-most dark triangle in the LDA imp. p-cov method corresponds to the data of one of the subjects, which shows the numerical issues described previously. However, these numerical issues do not apply to all poorly performing subjects. The second worst subject in the LDA imp. p-cov method (left-most dark square) for example shows performance gains with the novel method compared to the other methods.

Figure 8c shows the *BI_b*^*+*^ dataset with 480 epochs per virtual data subset. In this dataset, the overall performance of the LDA imp. p-cov method is slightly better than that of the runner-up LDA p-cov. Additionally, the performance of most subjects is very similar between the two methods, and only some subjects show a noticeable performance gain using the time-decoupled matrices. In the EEG data there we did not find any immediate indicator, such as artifacts or heavy noise, that indicates why that could be the case.

## Discussion

In this work we considered mostly the traditional two-class ERP oddball paradigms. As our proposed method improves the covariance matrix, it could also be applied to multi-class methods that require a covariance matrix estimation, e.g. multi-class LDA, given that the assumptions we made in “Time-decoupled Covariance Matrices” are fulfilled. Additionally, there are other BCI paradigms using different kind of signals. For error-related potentials (Dal Seno et al. 2010) and slow cortical potentials (Krauledat et al. 2004), our method should be applicable without any additional adaptations, except choosing the relevant time intervals. However, this needs to be confirmed in future work.

The transfer of our proposed method to oscillatory signals, such as steady state evoked potentials and event-related de-/synchronization in motor imagery, is not as straightforward. For these signals, usually the feature vector contains only spatial data from one single time interval. However, if features from multiple time intervals are used, our method should be applicable, given that our assumptions are fulfilled.

In this work, we only evaluated the classification of brain signals. In theory, our approach should be applicable to regression approaches. However, these have to be covariance-based and use a spatio-temporal covariance matrix. While these approaches exist, they typically use a spatial covariance matrix (Dähne et al. 2014) only, or use features in different frequency bands (Fatemi and Daliri 2020), which can violate the assumption of feature homoscedasticity over time.

For three subjects who were identified as negative performance outliers, we found that numerical instability can be caused by the proposed diagonal block replacement. Unfortunately, so far we found no indicators in the EEG signals, e.g. artifacts or heavy noise, which could predict if this rare problem will occur. In future work, we aim to determine the cause of the numerical instability from the data and how established approaches, e.g. regularization of the covariance matrix, can be implemented to obtain a well-conditioned matrix after the replacement operation.

Note that so far we only inspected the impact on LDA performance of the time-decoupling of the covariance matrix. While we can observe improved performances, it still is unclear whether this new covariance matrix is closer to the true underlying covariance matrix. In future work, we plan to run simulation studies to evaluate which matrix estimation technique, i.e. time-decoupling, shrinkage or the sample covariance matrix, is closer to the actual data generating covariance matrix.

We observed a performance benefit not only in these small datasets, but in most tested datasets of varying dimensionality and SNR level. Thus we are optimistic, that the applicability of our proposed novel method is not restricted to the domain of BCI. Instead, it could be valuable to apply it also to other data. Generally, any data which also has a spatio-temporal structure, and in which the spatial noise can be assumed to be constant with respect to time could profit from the proposed approach. Specific candidates are MEG and multi-electrode EMG recordings. Another possible application could be spatially distributed sensor networks that make use of identically constructed sensors.

We also found that using a linear kernel principal component analysis does not improve the performance of an LDA classifier for most datasets. This indicates that large dimensionality is not a primary issue in these datasets.

In ERP paradigms, the concrete ratio of target and non-target stimuli within chronological virtual data subsets depends on the used stimulus sequence. As in this work we focused on method development, we chose to use a stratified cross-validation scheme rather than a chronological cross-validation scheme which typically is preferred in the BCI domain. While the latter would have been closer to the final application, it could not guarantee that all folds have the same class ratios. This would have been a disadvantage for the comparison of methods, as we introduce another challenge into the benchmark, i.e. how well does a method handle differing class ratios in training and validation. In fact, for datasets recorded in our lab in which the generated stimulus sequence guarantees stratified folds, we observed, that the relative performance differences between the methods were nearly identical (data not shown), but the overall performance across methods dropped by up to 5 % points AUC, depending on the dataset.

Our proposed novel time-decoupling of the covariance has merit when using it to enhance the feature covariance matrix of an LDA classifier. We think our approach improves the usability of ERP-based BCIs due to several arguments, for which we found clear evidence in our extensive evaluation on multiple datasets.

First, our approach offers a simple, yet effective, way to improve the classification performance for very small datasets—a problem identified by multiple authors in the field of BCI, who emphasized the need for decoding algorithms to be able to handle the training with few data points.

Second, the new methods allows to shorten the required calibration time while still keeping the same classification performance—a quality that improves usability specifically for patient studies.

Third, our approach yields classification performances well above chance level even when using tiny (72 epochs) amounts of training data. This characteristic can make experimental parameter optimization feasible, as the classification performance can be estimated reliably even on very short EEG recordings.

Fourth, with increasing training data, the classification performance of our method converges to the performance of the regular LDA. Therefore, there is no harm done using our approach even when abundant training data is available or when it is unclear, if the size of the training data set is in the right range for profiting from the time-decoupled covariances.

Due to the aforementioned arguments, we would recommend BCI practitioners to use the proposed time-decoupled covariances for LDA as a first shot method, as it shows clear benefits over an ordinary shrinkage-regularized LDA for most ERP-classification scenarios.

## Conclusion

Using domain knowledge and exploiting the specific structure of the feature vectors in ERP classification paradigms, we propose a new way to estimate a covariance matrix that outperforms a shrinkage based covariance matrix, especially on small datasets. Our results could enable fast-adapting BCIs that require short calibration times. A possible application for our method is the tuning of stimulation parameters to an individual subject. Here, long recordings are not feasible and the information content of short recordings should be maximized to determine the optimal parameters.

## Information Sharing Statement

Results and most figures for the public datasets we used can be reproduced using the code available at https://github.com/jsosulski/time-decoupled-lda. The detailed instructions make it easy to obtain the results, especially when using the same system we used, i.e. Ubuntu 18.04 and python 3.6.9. The proposed improved classifier makes use of the widely used sklearn API and can be used as a drop-in replacement for other sklearn classifiers. The classifier is also available in the aforementioned repository. Public datasets used in this work are automatically downloaded using the provided code.

## Data Availability

All datasets used in this work are listed in Table 1. We used both *publicly* available data and restricted access data that was recorded in our lab and the subjects’ permission we collected did not include publishing raw EEG data. (See information sharing statement.)

## Footnotes

1 https://github.com/jsosulski/time-decoupled-lda

## Data Availability

The code that was used to produce the benchmark (on the public datasets) can be found in the above mentioned repository. The classifier implementation is also available there. As it follows the popular sklearn API, it can be used as a drop-in replacement for many python pipelines. (See information sharing statement.)

## References

[CR1] Allison BZ, Pineda JA (2006). Effects of SOA and flash pattern manipulations on ERPs, performance, and preference: implications for a BCI system. International Journal of Psychophysiology.

[CR2] Aricò P, Aloise F, Schettini F, Salinari S, Mattia D, Cincotti F (2014). Influence of p300 latency jitter on event related potential-based brain–computer interface performance. Journal of Neural Engineering.

[CR3] Barachant, A., & Congedo, M. (2014). A plug&play P300 BCI using information geometry. arXiv:14090107.

[CR4] Barachant, A., Bonnet, S., Congedo, M., & Jutten, C. (2010). Riemannian geometry applied to BCI classification. In *International conference on latent variable analysis and signal separation* (pp. 629–636): Springer.

[CR5] Bashashati H, Ward RK, Bashashati A (2016). User-customized brain computer interfaces using Bayesian optimization. Journal of Neural Engineering.

[CR6] Bishop, C.M. (2006). Linear models for classification. In *Pattern recognition and machine learning. chap 4* (pp. 179–220): Springer.

[CR7] Blankertz B, Lemm S, Treder M, Haufe S, Müller KR (2011). Single-trial analysis and classification of ERP components—a tutorial. NeuroImage.

[CR8] Dähne S, Meinecke FC, Haufe S, Höhne J, Tangermann M, Müller KR, Nikulin VV (2014). SPOc: a novel framework for relating the amplitude of neuronal oscillations to behaviorally relevant parameters. NeuroImage.

[CR9] Dal Seno, B., Matteucci, M., & Mainardi, L. (2010). Online Detection of p300 and Error Potentials in a Bci Speller. Computational intelligence and neuroscience, 2010. 10.1155/2010/307254 PMC2821756 20169142

[CR10] Farquhar J, Hill NJ (2013). Interactions between pre-processing and classification methods for event-related-potential classification. Neuroinformatics.

[CR11] Fatemi M, Daliri MR (2020). Nonlinear sparse partial least squares: an investigation of the effect of nonlinearity and sparsity on the decoding of intracranial data. Journal of Neural Engineering.

[CR12] Feess D, Krell MM, Metzen JH (2013). Comparison of sensor selection mechanisms for an ERP-based brain-computer interface. PloS One.

[CR13] Foodeh R, Khorasani A, Shalchyan V, Daliri MR (2016). Minimum noise estimate filter: a novel automated artifacts removal method for field potentials. IEEE Transactions on Neural Systems and Rehabilitation Engineering.

[CR14] Guger C, Daban S, Sellers E, Holzner C, Krausz G, Carabalona R, Gramatica F, Edlinger G (2009). How many people are able to control a P300-based brain–computer interface (BCI)?. Neuroscience Letters.

[CR15] Hoffmann U, Vesin JM, Ebrahimi T, Diserens K (2008). An efficient P300-based brain–computer interface for disabled subjects. Journal of Neuroscience Methods.

[CR16] Höhne, J., & Tangermann, M. (2012). How stimulation speed affects event-related potentials and BCI performance. In *2012 annual international conference of the IEEE engineering in medicine and biology society*. 10.1109/EMBC.2012.6346300 (pp. 1802–1805). 10.1109/EMBC.2012.6346300 23366261

[CR17] Holm, S. (1979). A simple sequentially rejective multiple test procedure. Scandinavian Journal of Statistics, 65–70.

[CR18] Hübner, D, Verhoeven, T., Schmid, K., Müller, K.R., Tangermann, M., & Kindermans, P.J. (2017). Learning from label proportions in brain-computer interfaces: online unsupervised learning with guarantees. *PloS one*, *12*(4). 10.1371/journal.pone.0175856 PMC5391120 28407016

[CR19] Hübner D, Verhoeven T, Müller KR, Kindermans PJ, Tangermann M (2018). Unsupervised learning for brain-computer interfaces based on event-related potentials: Review and online comparison. IEEE Computational Intelligence Magazine.

[CR20] Jayaram V, Barachant A (2018). MOABB: Trustworthy algorithm benchmarking for BCIs. Journal of Neural Engineering.

[CR21] Jayaram V, Alamgir M, Altun Y, Scholkopf B, Grosse-Wentrup M (2016). Transfer learning in brain-computer interfaces. IEEE Computational Intelligence Magazine.

[CR22] Kolkhorst, H., Tangermann, M., & Burgard, W. (2018). Guess what I attend: Interface-free object selection using brain signals. In *IEEE/RSJ international conference on intelligent robots and systems (IROS)*, (Vol. 2018 pp. 7111–7116): IEEE.

[CR23] Krauledat, M., Dornhege, G., Blankertz, B., Losch, F., Curio, G., & Muller, K.R. (2004). Improving speed and accuracy of brain-computer interfaces using readiness potential features. In *The 26th annual international conference of the IEEE engineering in medicine and biology society*, (Vol. 2 pp. 4511–4515): IEEE. 10.1109/IEMBS.2004.1404253 17271309

[CR24] Lal TN, Schroder M, Hinterberger T, Weston J, Bogdan M, Birbaumer N, Scholkopf B (2004). Support vector channel selection in BCI. IEEE Transactions on Biomedical Engineering.

[CR25] Ledoit O, Wolf M (2004). A well-conditioned estimator for large-dimensional covariance matrices. Journal of Multivariate Analysis.

[CR26] Lotte F, Congedo M, Lécuyer A, Lamarche F, Arnaldi B (2007). A review of classification algorithms for EEG-based brain–computer interfaces. Journal of Neural Engineering.

[CR27] Lotte F, Bougrain L, Cichocki A, Clerc M, Congedo M, Rakotomamonjy A, Yger F (2018). A review of classification algorithms for EEG-based brain–computer interfaces: a 10 year update. Journal of Neural Engineering.

[CR28] Musso, M., Bambadian, A., Denzer, S., Umarova, R., Hübner, D, & Tangermann, M. (2016). A novel BCI based rehabilitation approach for aphasia rehabilitation. In *Proceedings of the 6th international brain-computer interface meeting* (p. 104).

[CR29] Pedregosa F, Varoquaux G, Gramfort A, Michel V, Thirion B, Grisel O, Blondel M, Prettenhofer P, Weiss R, Dubourg V, Vanderplas J, Passos A, Cournapeau D, Brucher M, Perrot M, Duchesnay E (2011). Scikit-learn: Machine learning in Python. Journal of Machine Learning Research.

[CR30] Riccio A, Simione L, Schettini F, Pizzimenti A, Inghilleri M, Olivetti Belardinelli M, Mattia D, Cincotti F (2013). Attention and P300-based BCI performance in people with amyotrophic lateral sclerosis. Frontiers in Human Neuroscience.

[CR31] Rivet B, Souloumiac A, Attina V, Gibert G (2009). xDAWN algorithm to enhance evoked potentials: application to brain–computer interface. IEEE Transactions on Biomedical Engineering.

[CR32] Russakovsky O, Deng J, Su H, Krause J, Satheesh S, Ma S, Huang Z, Karpathy A, Khosla A, Bernstein M (2015). Imagenet large scale visual recognition challenge. International Journal of Computer Vision.

[CR33] Rutkowski TM, Mori H (2015). Tactile and bone-conduction auditory brain computer interface for vision and hearing impaired users. Journal of Neuroscience Methods.

[CR34] Sannelli C, Dickhaus T, Halder S, Hammer EM, Müller KR, Blankertz B (2010). On optimal channel configurations for SMR-based brain–computer interfaces. Brain Topography.

[CR35] Schölkopf, B., Smola, A., & Müller, K.R. (1997). Kernel principal component analysis. In *International conference on artificial neural networks* (pp. 583–588): Springer.

[CR36] Schreuder M, Blankertz B, Tangermann M (2010). A new auditory multi-class brain-computer interface paradigm: Spatial hearing as an informative cue. PLOS One.

[CR37] Sellers, E.W., Arbel, Y., & Donchin, E. (2012). BCIs that use P300 event-related potentials. In *Brain-computer interfaces: principles and practice* (p. 215): Oxford University Press.

[CR38] Sosulski, J., & Tangermann, M. (2019). Spatial filters for auditory evoked potentials transfer between different experimental conditions. In *Proceedings of the 8th Graz brain-computer interface conference*, (Vol. 2019 pp. 273–278).

[CR39] Srinivasan, R. (2012). Acquiring brain signals from outside the brain. In *Brain-computer interfaces: principles and practice. chap 6* (pp. 105–122).

[CR40] Sugi M, Hagimoto Y, Nambu I, Gonzalez A, Takei Y, Yano S, Hokari H, Wada Y (2018). Improving the performance of an auditory brain-computer interface using virtual sound sources by shortening stimulus onset asynchrony. Frontiers in Neuroscience.

[CR41] Van Veen, G., Barachant, A., Andreev, A., Cattan, G., Rodrigues, P.C., & Congedo, M. (2019). Building Brain Invaders: EEG data of an experimental validation. arXiv:190505182.

[CR42] Wilcoxon F, Katti S, Wilcox RA (1970). Critical values and probability levels for the Wilcoxon rank sum test and the Wilcoxon signed rank test. Selected Tables in Mathematical Statistics.

[CR43] Winkler I, Brandl S, Horn F, Waldburger E, Allefeld C, Tangermann M (2014). Robust artifactual independent component classification for BCI practitioners. Journal of Neural Engineering.

[CR44] Wolpaw JR, Birbaumer N, McFarland DJ, Pfurtscheller G, Vaughan TM (2002). Brain–computer interfaces for communication and control. Clinical Neurophysiology.

